# Automatic Registration of Terrestrial Laser Scanning Point Clouds using Panoramic Reflectance Images

**DOI:** 10.3390/s90402621

**Published:** 2009-04-15

**Authors:** Zhizhong Kang, Jonathan Li, Liqiang Zhang, Qile Zhao, Sisi Zlatanova

**Affiliations:** 1 Faculty of Aerospace Engineering, Delft University of Technology / Kluyverweg 1, 2629 HS Delft, The Netherlands; 2 School of Land Science and Technology, China University of Geosciences / Beijing 100083, China; 3 Department of Geography and Environmental Management, University of Waterloo / Waterloo, Ontario N2L 3G1, Canada; E-Mail: junli@uwaterloo.ca; 4 Research Centre for Remote Sensing and GIS, School of Geography, Beijing Normal University / Beijing 100875, P.R. China; E-Mail: zhanglq@bnu.edu.cn; 5 GNSS Research Centre, Wuhan University / Wuhan 430079, Hubei Province, P.R. China; E-Mail: zhaoql@whu.edu.cn; 6 OTB Research Institute for Housing, Urban and Mobility Studies, Delft University of Technology / Jaffalaan 9, 2628 BX Delft, The Netherlands; E-Mail: s.zlatanova@tudelft.nl

**Keywords:** Point cloud, Registration, LIDAR, Terrestrial laser scanning, Automation, Image Matching

## Abstract

This paper presents a new approach to the automatic registration of terrestrial laser scanning (TLS) point clouds using panoramic reflectance images. The approach follows a two-step procedure that includes both pair-wise registration and global registration. The pair-wise registration consists of image matching (pixel-to-pixel correspondence) and point cloud registration (point-to-point correspondence), as the correspondence between the image and the point cloud (pixel-to-point) is inherent to the reflectance images. False correspondences are removed by a geometric invariance check. The pixel-to-point correspondence and the computation of the rigid transformation parameters (RTPs) are integrated into an iterative process that allows for the pair-wise registration to be optimised. The global registration of all point clouds is obtained by a bundle adjustment using a circular self-closure constraint. Our approach is tested with both indoor and outdoor scenes acquired by a FARO LS 880 laser scanner with an angular resolution of 0.036° and 0.045°, respectively. The results show that the pair-wise and global registration accuracies are of millimetre and centimetre orders, respectively, and that the process is fully automatic and converges quickly.

## 1. Introduction

Terrestrial laser scanning (TLS) has been proven effective in urban mapping for applications ranging from as-built documentation to the three-dimensional (3D) reconstruction of architectural details and building facades. However, one of the biggest problems encountered while processing the scans is TLS point cloud registration in which rigid transformation parameters (RTPs) are determined in order to bring one dataset into alignment with the other. RTPs are computed by common points, obtained by correspondence tracking, in scenes from different viewpoints. Commercial software typically uses separately scanned markers that can be automatically identified as corresponding points. Several surface matching algorithms have been proposed to avoid the use of artificial markers. The most popular method is the iterative closest point (ICP) algorithm developed by Besl and McKay [1]. Several improvements to the ICP algorithm have been proposed, such as the iterative closest compatible point (ICCP) [2] and the iterative closest points using invariant features (ICPIF) [3]. The ICP algorithm requires a good first approximation in order to converge to a global minimum. However, even if there is considerable overlap, convergence to a global minimum is not guaranteed. The ICP algorithm can also be computationally intensive and time consuming in its search for conjugate points in overlapping scans [4].

In recent years, a great deal of effort has been devoted to developing approaches based on segmenting TLS point clouds and thereby matching extracted primitives. Primitives are derived from point clouds and are matched in a semi-automatic or fully automatic way (e.g., [5,6]). Primitive-based matching methods can be very successful in the case of well-determined shapes (such as pipe installations or single buildings). However, as discussed in Dold and Brenner [7], for the most part there exist only two prevalent directions of normal vectors (of planar patches) along urban streets, namely, perpendicular to the facades of buildings and perpendicular to the streets. In this case, the translation parameters are weakly determined, due to the lack of a third perpendicular plane. The rotation parameters can still be derived because they are not affected by the lack of a third plane.

Today, most TLS manufacturers offer the option of a high-resolution digital camera mounted on the scanner for users to capture digital imagery while TLS point clouds are collected, so as to generate photorealistic 3D object and scene models. The generally higher resolution of the optical images and the well-established image processing algorithms offer attractive possibilities for automatically aligning the TLS point clouds. Several methods can be found in the literature and are referred to as image-based registration (IBR). For example, Wendt [8] used the stochastic optimisation principle of simulated annealing (also known as the metropolis algorithm) to match certain patterns in discrete orthoimages, to thereby extract features from the images, and finally to fit them into planes using point clouds. Dold and Brenner [7] employed image information to verify uncertain translation parameters, which are computed by planar patches extracted from point clouds. Seo *et al.* [9] used distinctive image features for point cloud registration. Their approach relies on modifying the 2D image features using the 3D point cloud to achieve greater invariance to projective distortion than is generally associated with existing 2D image feature descriptors. Al-Manasir and Fraser [10] proposed a registration method using photogrammetric orientation of images acquired by a scanner-mounted camera. Since images are registered to the TLS scans, the point clouds from each scanner position can be registered using the relative orientation between each pair of registered images. However, since coplanarity is not expected, panoramic reflectance images are not applied in the relative orientation model that they used, which is based on coplanarity. Barnea and Filin [11] presented a registration scheme that matches the extracted features with 2D optical images using the scale invariant feature transform (SIFT), followed by computing the actual transformation between the scans in 3D space using the RANSAC (RANdom SAmpling Consensus) algorithm developed by Fischler and Bolles [12]. Similar to this method, Barnea and Filin [13] developed a key-point based autonomous registration method using range images that also uses the 3D Euclidean distance between key-points as matched entities to identify correspondence.

Our earlier work described an algorithm for automatically registering TLS point clouds using reflectance images [14,15]. The algorithm takes advantage of the pixel-to-point correspondence that is inherent to the reflectance images and thus circumvents camera calibration and camera to scanner registration in the case of point cloud registration using optical images. However, the Moravec normal corner detector [16], which is used in [14, 15], is not expected to be useful in the case of a panoramic stereo pair. As it conforms only to focal plane array optical cameras, it is quite difficult to make any assumptions about the set of possible correspondences for a given feature point extracted from a panoramic image using the normal corner detector.

Many applications require more than two scans of any one object or scene. The global registration of multiple scans is more difficult because of the large nonlinear search space and the huge number of raw TLS data involved. Several useful approaches to this problem have been proposed in recent years [17, 18, 19] by means of incrementally registering views against a growing global union of viewpoints. Bergevin *et al.* [20] presented an algorithm that considers the network of views as a whole and minimises the registration errors of all views simultaneously. Inspired by that work, Benjemaa and Schmitt [21] extended the pair-wise registration based on a multi-z-buffer technique to a global registration. They applied rigid transformations, such that it became possible to transform each moving surface immediately after its rigid transformation had been estimated. Similarly, Sharp *et al.* [22] proposed an analytical method to solve for global registration parameters that involves building a graph to describe the relationship between neighbouring views. This approach then decomposes the graph into basis cycles so that the nonlinear optimisation problem can be solved over each basis cycle in a closed form. Hu *et al.* [23] built a topological graph to determine the best registration path of all range scans. Stoddart and Hilton [24] identify pair-wise correspondences between points in all views and then iteratively minimise correspondence errors over all views using a descent algorithm. This basic technique was extended by Neugebauer [25] and Eggert *et al.* [26] using a multiresolution framework, surface normal processing, and boundary point processing. Williams and Bennamoun [27] suggested a further refinement by including individual covariance weights for each point. There is currently no consensus as to the best approach for solving the global registration problem. Besides, additional sensors, e.g., Global Navigation Satellite System (GNSS), compasses, and tilt sensors, are now often combined with TLS instruments to help partly solve or reduce the global registration problem.

This paper extends our previous work [14, 15, 28] and presents a new approach to automatic IBR that searches for corresponding points based on the projected panoramic reflectance imagery of 360° TLS point clouds. Unlike ICP and primitive-based methods, our approach maps a 3D point cloud into a 2D image, which greatly simplifies the registration. This simplification is achieved in two ways: the 3D correspondence problem is reduced to 2D, and thus the data used in the registration are remarkably reduced. In most IBR approaches that use optical images, a camera calibration and camera-to-scanner registration are required, both of which may be error-prone. Moreover, the resolution differences between point clouds and optical images also result in errors of pixel-to-point correspondence. Our method avoids these problems because it uses the reflectance imagery that is created directly from scans (including the angular coordinates and reflectance value of each 3D point). In addition, the image point correspondence and our computation of the RTPs are integrated into an iterative process that allows for registration optimisation. Compared with our earlier work [14, 15], we now use the SIFT method, instead of the Moravec normal corner detector, which makes correspondence tracking feasible in the case of a panoramic stereo pair. We note that SIFT can provide robust matching across a substantial range of affine distortions, changes in 3D viewpoint, additions of noise, and changes in illumination. In the case of global registration, assuming a formed ring scheme, the circular self-closure constraint is deduced to circumvent the influence of correspondence error, which in turn helps us arrive at a global optimisation.

The remainder of this paper is organised as follows. The following two sections detail the proposed approach, including pair-wise registration and global registration, respectively. Section 4 discusses our test results, after which we offer our conclusions, together with suggestions for further research.

## 2. Pair-wise Registration

In this paper, we distinguish between three types of correspondences, namely, pixel-to-pixel, pixel-to-point, and point-to-point. Pixel-to-pixel correspondence is between image pixels of two overlapping images, while pixel-to-point correspondence involves image pixels and 3D points from a TLS scan. The latter is known when either reflectance imagery is employed or optical images are available. The TLS units with a built-in digital camera usually provide this correspondence. Alternatively, it can be computed by knowing the camera calibration and orientation parameters. Recent research (e.g., [29, 30]) has described methods to find the pixel-to-point correspondence between images taken from a high resolution camera independent of the scanner. Point-to-point correspondence involves 3D points from two overlapping TLS scans.

As pixel-to-point correspondence is inherent to reflectance images, we only explain how the reflectance images are constructed and elaborate on the two other processes, namely, pixel-to-pixel and point-to-point correspondence. Image matching (which allows us to establish the pixel-to-pixel correspondence) has been widely explored in photogrammetry and computer vision communities for years. Many mature algorithms for automatic pixel-to-pixel correspondence have been proposed to date (e.g., [31–34]). Using such algorithms and knowing the pixel-to-point correspondence, we can register TLS point clouds fully automatically. Once the correspondences are established, an ICP algorithm can be used to improve the registration accuracy. However, this can be very computationally intensive since a 360° scan contains millions of points. In this study, an iterative process is employed to track additional correspondences and to obtain a homogeneous distribution of corresponding point pairs in order to achieve high registration accuracy.

Throughout this paper, in identifying a pair of scans as being co-registered, the scan that defines the coordinate system is termed the *fixed scan,* while the scan to be transformed into this coordinate system is denoted the *moving scan*. Consequently, the reflectance images created from these scans inherit the same notation; i.e., they are named the *fixed image* and the *moving image*, respectively.

### 2.1. Registration Process

**Figure 1.**
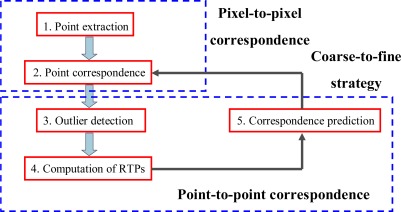
Flowchart of our pair-wise registration strategy.

In general, the registration process requires RTPs. Our method computes RTPs using a two-stage iterative process that includes both pixel-to-pixel and point-to-point correspondence (see Figure 1).

The entire iterative process consists of five steps: (1) point extraction, (2) point correspondence, (3) outlier detection, (4) computing RTPs, and (5) correspondence prediction. The first two steps are performed on the reflectance images and output all possible candidate points for the subsequent computation of RTPs. The subsequent three steps are performed on the TLS points. 3D corresponding points are taken on the basis of the known pixel-to-point correspondence. When only image information is used, some correspondences may be incorrect; outlier detection should be implemented to remove such incorrectly matched points. The RTPs computed from the 3D corresponding points at the beginning may remain after outlier detection. Generally speaking, most of the corresponding points that exhibit repetitive patterns in those regions are likely to be falsely accepted by the image-based matching method. Thus, they should be removed after outlier detection so that the distribution of corresponding point pairs in the overlapping area is unlikely to be homogeneous. The iterative process can track out the correct correspondences. Using the RTPs computed in earlier iterations, 2D correct correspondences on the moving image can be better determined, which results in an increased number of matched points. Then, the new matches can be added into the computation of new transformation parameters for the subsequent iteration. The iterative process continues until the transformation parameters reach the predefined accuracy threshold.

### 2.2. Generation of Reflectance Images

**Figure 2.**
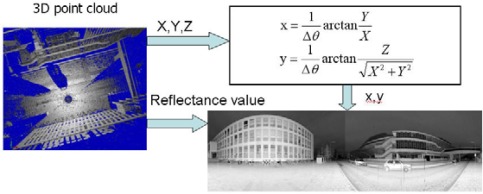
Procedure to construct the reflectance image from a 3D point cloud.

In general, each instrument observation comprises four values, namely, horizontal and vertical angles, range and reflectance value. 3D points are calculated as a combination of the horizontal and vertical angles of the laser beam plus the measured distance. The reflectance imagery can be created directly from scans using the angular coordinates and reflectance value of each point (see Figure 2). Equation 1 parameterises the mapping from a point to its corresponding point on the reflectance image:

(1) $$\begin{array}{c} x=\frac{1}{\Delta \theta }\text{arctan}\frac{Y}{X}\\ y=\frac{1}{\Delta \theta }\text{arctan}(\frac{Z}{\sqrt{X^{2}+Y^{2}}}) \end{array}$$

where *x*, *y* are the image coordinates of a point, whose ranges are determined by the FOV and angular resolution of the scan, *X*,*Y*,*Z* are the 3D coordinates, and Δ*θ* is the angular resolution of the scan.

Concerning the TLS scans, the angular resolution Δ*θ* and scanning angle range are pre-defined before scanning, so they can be regarded as inherent parameters of the scan. The 3D coordinates can be converted into the reflectance image coordinate system that originates at the left bottom/top corner of the image in terms of the scanning angle. Therefore, the pixel-to-point correspondence is inherent to the reflectance images. Moreover, the panoramic reflectance imagery can be theoretically generated, in terms of the angular coordinate and reflectance value of each point, directly from TLS scans. Figure 2 shows a vertical discontinuity line in the centre of the reflectance image, which is due to the scanning mechanism of the FARO LS 880. During one revolution of the mirror, the FARO LS 880 scans vertically from top to bottom at one side of the machine, and then from bottom to top at the other side. It then rotates horizontally and scans the next two columns of the image. At the end, therefore, after finishing a complete 360 degree scan, half of the scene has been scanned from top to bottom and the other half has been scanned from bottom to top. These two halves meet in the middle. If they do not fit exactly (for example if the scanner moves slightly during the scanning), one will see an edge.

### 2.3. Pixel-to-Pixel Correspondence

The pixel-to-pixel correspondence is critical for our proposed method. The image matching algorithm employed to find corresponding points between the adjacent images is explained below.

Since 360° reflectance images are used, it is generally quite difficult to make any assumption regarding the set of possible correspondences for a given feature point that can be extracted by normal corner detectors, which merely conform to focal plane array optical cameras. We therefore use the SIFT method [33], as it can provide robust matching across a substantial range of affine distortions, changes in 3D viewpoints, additions of noise, and changes in illumination. The SIFT method has been previously used in image-based registration (e.g., [9, 11]), but we are not aware of its application to date in the realm of panoramic reflectance imagery.

Similar to Lowe [33], we implemented the following steps to generate a set of distinctive invariant features (points):

1. Scale-space extrema detection: employing a difference-of-Gaussian function to identify potential points of interest that are invariant to scale and orientation,
2. Key-point localisation: fitting an analytical model (mostly in the form of a parabola) at each candidate location to determine the location and scale,
3. Orientation assignment: assigning one or more orientations to each key-point location based on local image gradient directions, and
4. Key-point descriptor: measuring the local image gradients at the selected scale in the region around each key-point.

When using reflectance images, gray values depend heavily on the viewpoint and the angle of illumination. Moreover, the gradients may vary between different scans. However, the SIFT method can cope with these problems by reducing the effects of variable illumination conditions. First, the descriptor vector is normalised to unit length so that the image contrast change is cancelled by vector normalisation. A brightness change in which a constant is added to each image pixel will not affect the gradient values, as they are computed from pixel differences. Therefore, the key-point descriptor is invariant to affine changes in illumination. Variable non-linear illumination can cause a change in relative magnitudes for some gradients, but is less likely to affect the gradient orientations. Therefore, the influence of large gradient magnitudes is reduced by thresholding the values in the unit feature vector, and then renormalising to unit length. This means that matching the magnitudes for large gradients is no longer important, and the distribution of orientations carries greater weight.

To identify correspondences, the invariant descriptor vector for the key-point is given as a list of 128 integers in the range [0,255]. Correspondences can generally be tracked out among images. Key-points from a given image can be matched to those from previous images by simply looking for the descriptor vector that exhibits the closest Euclidean distance among all vectors from previous images. To identify matches, we use Lowe’s [33] method to find the two nearest neighbours of each key-point from the moving image. A match is identified if the ratio of the distance from the closest neighbour to that of the second closest is less than a predefined threshold. The predefined threshold can be increased to select more matches or decreased to select only the most reliable match. In addition, the *k*-dimensional tree (*k*-D tree) [35] is used to speed up the search. The *k*-D tree is a space-partitioning data structure used to organise points in *k*-dimensional space. It is very useful when a multidimensional search key (e.g., range search and nearest neighbour search) is involved.

This approach can be used to easily identify false matches from the panoramic reflectance images of buildings, as building facades are likely to exhibit repetitive patterns. The rigid geometric invariance derived from TLS point clouds is used to prune false correspondences in the next step, namely, point-to-point correspondence.

### 2.4. Point-to-Point Correspondence

In practice, it is impossible to correctly match all 3D correspondences. Most likely, some incorrect corresponding points also get inadvertently accepted. A rigid geometric invariance is used to detect and remove these false correspondences.

#### 2.4.1 Outlier Detection

**Figure 3.**
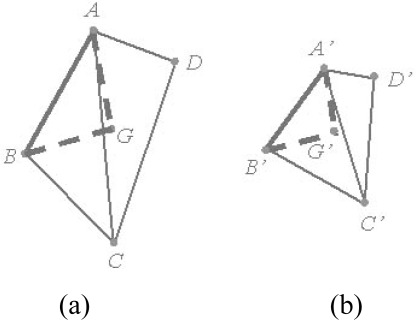
Four corresponding TLS points from two overlapping scans. The distance between the two points in Scan (a) should equal the distance between the two corresponding points in Scan (b), i.e., *S*_AB_ = *S*_A′B′_. *G* and *G′* are the gravity points of points *A*, *B*, *C*, *D* and *A′*, *B′*, *C′*, *D′*, respectively. The gravity points are used to verify those legitimate correspondences that are disqualified because they are surrounded by wrong matches.

Figure 3 illustrates the two overlapping TLS scans. A, B, C, and D are 3D points from TLS Scan (a), while A′, B′, C′, and D′ are corresponding points from TLS Scan (b). Since the Euclidean distance between the two corresponding point pairs is invariant in the local coordinate system under the assumption of an ideal sensor and identical sampling of data points, the distance between the two points in Scan (a) should equal the distance between the two corresponding points in Scan (b), i.e., *S*_AB_ = *S*_A′B′_.

However, this verification process may increase the computation time considerably. To avoid this, we construct a Triangulated Irregular Network (TIN) using Delaunay triangulation and use the relations between the points in the triangles to determine the distances. The TIN model is selected because of its simplicity and computational economy. The 2D Delaunay triangulation is computed in the image plane and then mapped to TLS point clouds. As the TIN is constructed to verify distance invariance between correspondences, we merely need to construct the TIN model for 3D points in one scan and replicate it in the other using the corresponding points. Consequently, only those point-pairs that are connected in the TIN model are verified for distance invariance.

The distance between the two points in Scan (a) should equal the distance between the two corresponding points in Scan (b), i.e., *S*_AB_ = *S*_A′B′_ (Figure 3). However, it is practically impossible for *S_AB_* to exactly equal *S_A′B′_* due to the location error of 3D points. Therefore, we need to estimate the tolerable error for distance invariance. In the work of Barnea and Filin [13], this value is empirically acquired. However, as the distance invariance error is variant and related to the two corresponding pairs, the tolerable value should be self-adaptive. The equation for the distance difference between 3D points A, B and their corresponding points A′, B′ is:

(2) $$\Delta S=\sqrt{{\left(X_{A}-X_{B}\right)}^{2}+{\left(Y_{A}-Y_{B}\right)}^{2}+{\left(Z_{A}-Z_{B}\right)}^{2}}-\sqrt{{\left(X_{A^{\prime }}-X_{B^{\prime }}\right)}^{2}+{\left(Y_{A^{\prime }}-Y_{B^{\prime }}\right)}^{2}+{\left(Z_{A^{\prime }}-Z_{B^{\prime }}\right)}^{2}}$$

where *ΔS* is the distance difference, and (*X_A_*, *Y_A_*, *Z_A_*), (*X_B_*, *Y_B_*, *Z_B_*), (*X_A′_*, *Y_A′_*, *Z_A′_*) and (*X_B′_*, *Y_B′_*, *Z_B′_*) are respectively the 3D coordinates of points *A*, *B*, *A′* and *B′*.

The error of the distance invariance σ_Δ*S*_ can be estimated using the error propagation law in light of the location error of the two corresponding point pairs. Because the location error of each point is independent, the error estimation can be obtained by:

(3) $$\sigma_{\Delta S}^{2}=C_{Y_{A}}D_{(\mathrm{XYZ})A}C_{Y_{A}}{}^{T}+C_{Y_{B}}D_{(\mathrm{XYZ})B}C_{Y_{B}}{}^{T}+C_{Y_{A^{\prime }}}D_{(\mathrm{XYZ})A^{\prime }}C_{Y_{A^{\prime }}}{}^{T}+C_{Y_{B^{\prime }}}D_{(\mathrm{XYZ})B^{\prime }}C_{Y_{B^{\prime }}}{}^{T}$$

where $\sigma_{\Delta S}^{2}$ is the variance of the distance invariance, *D*_(*XYZ*)*i*_ is the variance matrix of point *i*, *i* = *A*, *B*, *A′* and *B′,* and

(4) $$C_{Y_{A}}=\frac{1}{S_{\mathrm{AB}}}\left[\begin{array}{ccc} X_{A}-X_{B} & Y_{A}-Y_{B} & Z_{A}-Z_{B} \end{array}\right],C_{Y_{B}}=-C_{Y_{A}}$$

(5) $$C_{Y_{B^{\prime }}}=\frac{1}{S_{A^{\prime }B^{\prime }}}\left[\begin{array}{ccc} X_{A^{\prime }}-X_{B^{\prime }} & Y_{A^{\prime }}-Y_{B^{\prime }} & Z_{A^{\prime }}-Z_{B^{\prime }} \end{array}\right],C_{Y_{A^{\prime }}}=-C_{Y_{B^{\prime }}}$$

According to the error propagation law, the variance matrix of point *i* is derived from the location error of point *i*, which depends on the accuracy of the TLS system. Boehler [36] suggested that the accuracy of the TLS system consists of the angular and range accuracy, resolution, edge effects and more. Since laser scanning is a discrete sampling technique, the error of distance invariance mainly arises from the different samplings of the two scans. Moreover, the localisation error of the SIFT key-point may also influence the distance invariance. As the SIFT method is expected to localise the key-point to sub-pixel accuracy and because the pixel size of the reflectance image corresponds to the angular resolution of the scan, the errors of both sampling difference and key-point localisation can be modelled by taking into account the angular resolution for error propagation. Therefore, in this paper we take into account the angular resolution in the locational error estimation as well as the angular and range accuracies. Lichti [37] presented the systematic error models of the AM-CW TLS system (e.g., Faro 880). Those models are empirically determined to evaluate the accuracies of instrumental observation (e.g., range, horizontal and vertical angles). As our purpose here is to evaluate the tolerable error of the distance invariance, instead of investigating systematic error models, we only consider the range error σ*_R_*, the horizontal angular error σ*_θ_*, the vertical angular error σ*_φ_* and the angular resolution as invariant and independent for every point. Therefore, the variance matrix of point *i D*_(*XYZ*)*i*_ is computed in terms of σ*_R_*, σ*_θ_*, σ*_φ_* and the angular resolution, according to the error propagation law.

Three times the error of distance invariance σ_Δ*S*_ is chosen as a threshold to determine the correct correspondence, so Equation 2 can be written as

(6) $$|S_{\mathrm{AB}}-S_{A\prime B\prime }|<{3\sigma }_{\Delta S}$$

In Equation 3, σ_Δ*S*_ is a variant and is related to each of the two corresponding pairs. Therefore, the chosen threshold is self-adaptive, instead of a constant. If the above condition is satisfied, those two point pairs are said to be corresponding. Otherwise, there should be an outlier among the point pairs. According to Equation 6, however, we cannot determine which point pair is an outlier, or whether both of them are.

If the four point pairs shown in Figure 3 correspond, respectively, the gravity point pair *G* and *G′* of those point pairs should correspond as well. Accordingly, we select the point pairs consistent with Equation 6 to compute the gravity point pair. The distances are computed between the gravity point pair and those point pairs not satisfying Equation 6. The outliers should be those point pairs with differences between corresponding distances that are no smaller than 3σ_Δ*S*_.

#### 2.4.2. Computation of Transformation Parameters

The coordinates of each single TLS scan are recorded in a local coordinate frame defined by the instrument. Using the corresponding points detected in the previous step, it is possible to compute transformation parameters between different coordinate frames and thus register the two point clouds. The least-squares adjustment for the absolute orientation in photogrammetry [38] is used to solve least-squares optimised values of RTPs.

We note that, after outlier detection, the incorrectly matched points have been removed and RTPs can be computed only with the correct ones. However, the outlier detection may remove so many points that the remaining correspondences are unlikely to be uniformly distributed in the overlapping areas. Therefore, RTPs determined from these cannot be considered final. To improve them, more matching-appropriate points must be found. We use an iterative process to seek candidate points from amongst the feature points already extracted in the step pixel-to-pixel correspondence.

#### 2.4.3. Correspondence Prediction

Using the initial RTPs and inherent pixel-to-point correspondence, the feature points in the fixed reflectance image can be projected onto the moving image for the purpose of correspondence prediction. As presented earlier, based on the image coordinates (*x*, *y*) of a feature point in the fixed image, we can acquire the coordinates (*X*, *Y*, *Z*) of the corresponding 3D point of the fixed scan. The coordinates (*X’*, *Y’*, *Z’*) in the moving scan can be calculated from (*X*, *Y*, *Z*) using initial RTPs. The image coordinates (*x’*, *y’*) corresponding to (*X’*, *Y’*, *Z’*) represent the expected position of the corresponding point in the moving image. Thereafter, a certain region centred at (*x’*, *y’*) is determined to track the exact corresponding point. Correspondences are identified again based on predicted positions with a smaller search region and outlier detection is implemented. This means that more points from the extracted feature points on the fixed image can be correctly matched. New values of RTPs are computed across the entire set of old and newly matched points.

Each iteration process accordingly consists of four steps (see Figure 1): corresponding point prediction (Step 5) using RTPs computed from the previous iteration, identifying correspondence (Step 2), outlier detection (Step 3) and transformation parameter computation (Step 4). This iterative process ensures the matching of larger number of points and a homogeneous distribution of corresponding points, which leads to improved RTPs. The iterative process continues until the root-mean-square (RMS) error of the transformation parameter computation satisfies a given threshold. This threshold is in the range of millimetres and is determined with respect to the angular resolution, angular and range accuracy of the scanner. This iterative process completes our procedure and the final RTPs can then be used to register the two scans.

## 3. Global Registration

In general, global registration considers the network of views as a whole and minimises the registration errors across all views simultaneously. The network topology can be star-shaped (e.g., [20]), a cycle (e.g., [22]) or any other graph to determine the best registration path of all range scans. In many cases, one can logically design ring schemes for scanning targets. Accordingly, we develop a global registration algorithm under the assumption of a formed ring scheme. As explained in the previous sections, the pair-wise registration is based on the point correspondences tracked from reflectance images, and thus the global registration of a long chain of scans can be seen as a problem that relates to photogrammetric triangulation of a single strip, which in turn relies on the bundle adjustment of correspondences. Moreover, the closure constraint can be drawn from the ring scheme and imposed in the bundle adjustment as a solution to the optimisation problem. The closure constraint is normally formed under the last-to-first paradigm, which is unfortunately expected to introduce a correspondence error. Therefore, our global registration algorithm extends the closure constraint into a circular and self-closure paradigm.

**Figure 4.**
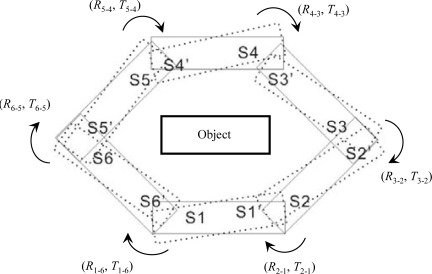
Circular self-closure constraint. The solid rectangle S*i* denotes the real scan, and the dotted version S*i*’ is computed from S*i* using six sets of RTPs (*i* = 1,…,6). Under the proposed adjustment, S*i* is fixed, and the six sets of RTPs are adjusted to minimise the sum of the squares of the disagreements between S*i* and S*i* ’.

Given six scans of a 3D object (see Figure 4), we choose without a loss of generality S1 as a *master* and the others as *slaves*. The reference frame of S1 is then defined as the world reference frame. Thus, the registration process must determine five rigid transformations, (*R*_2-1_, *T*_2-1_), (*R*_3-2_, *T*_3-2_),*…*, (*R*_6-5_, *T*_6-5_), corresponding to the motion of each slave into the master reference frame. The last-to-first closure constraint can be expressed as follows:

(7) $$\left[\begin{array}{c} X_{1}\\ Y_{1}\\ Z_{1} \end{array}\right]=R_{1}R_{2}\cdots R_{m-1}\left[\begin{array}{c} X_{m}\\ Y_{m}\\ Z_{m} \end{array}\right]+\left[\begin{array}{c} T_{1,X}\\ T_{1,Y}\\ T_{1,Z} \end{array}\right]+R_{1}\left[\begin{array}{c} T_{2,X}\\ T_{2,Y}\\ T_{2,Z} \end{array}\right]+R_{1}R_{2}\left[\begin{array}{c} T_{3,X}\\ T_{3,Y}\\ T_{3,Z} \end{array}\right]+\cdots +R_{1}R_{2}\cdots R_{m-2}\left[\begin{array}{c} T_{m-1,X}\\ T_{m-1,Y}\\ T_{m-1,Z} \end{array}\right]$$

where (*X*_1_, *Y*_1_, *Z*_1_) and (*X_m_*, *Y_m_*, *Z_m_*) are the correspondence coordinates in the first and last scans, respectively; (*T_i,X_*, *T_i,Y_*, *T_i,Z_*) represent the translation from scan *i*+1 to *i*; and *R*_i_ is the rotation from *i*+1 to *i*.

Equation (7) represents a rigid body transformation. Besides the scans acquired by the same scanner, Equation (7) can also be applied to those obtained from different sources and at different times since the laser range data establishes absolute scale so that the scale factor is unity.

Intuitively, the point coordinates (*X*_m_, *Y*_m_, *Z*_m_) in the last scan *m* are ideally transformed into their corresponding (*X*_1_, *Y*_1_, *Z*_1_) in the first scan using m−1 rigid transformations. The discord of this transformation process in practical terms is referred to as the closure error. However, in the case of Equation 7 it comprises not only transformation error, but also correspondence error, which is not expected to decrease during the bundle adjustment process. As a result, the closure constraint expressed by Equation 7 is not error-free in the ideal case.

To tackle this imperfection, we now extend the closure constraint into a self-closure form. An additional transformation from S1 to S6 is therefore computed as (*R*_1-6_, *T*_1-6_). As shown in Figure 4, (*R*_1-6_, *T*_1-6_) enables the first self-closure scan S1, which can be expressed as follows:

(8) $$\left[\begin{array}{c} X_{1}\\ Y_{1}\\ Z_{1} \end{array}\right]=R_{1}R_{2}\cdots R_{m}\left[\begin{array}{c} X_{1}\\ Y_{1}\\ Z_{1} \end{array}\right]+\left[\begin{array}{c} T_{1,X}\\ T_{1,Y}\\ T_{1,Z} \end{array}\right]+R_{1}\left[\begin{array}{c} T_{2,X}\\ T_{2,Y}\\ T_{2,Z} \end{array}\right]+R_{1}R_{2}\left[\begin{array}{c} T_{3,X}\\ T_{3,Y}\\ T_{3,Z} \end{array}\right]+\cdots +R_{1}R_{2}\cdots R_{m-1}\left[\begin{array}{c} T_{m,X}\\ T_{m,Y}\\ T_{m,Z} \end{array}\right]$$

Compared with Equation 7, (*X_m_*, *Y_m_*, *Z_m_*) is replaced by (*X*_1_, *Y*_1_, *Z*_1_) and accordingly one more transformation (*R*_m_, *T*_m_) is added, which means that after m rigid transformations (*X*_1_, *Y*_1_, *Z*_1_) maps back to itself in the ideal case. The closure constraint is error-free. In practical terms, the closure error can reach least squares after a bundle adjustment that merely consists of parameter errors. In addition, the self-closure form can be further extended to a circular one, as clearly the transformation ring ((*R*_2-1_, *T*_2-1_),*…*, (*R*_1-6_, *T*_1-6_)) allows each of the six scans to form a self-closure model (see Figure 4). The self-closure error of scan *i* can be visualised by the disagreement between the solid rectangle S*i* and the dotted one S*i*’ (*i* = 1,…,6). The bundle adjustment allows self-closure errors of all scans to simultaneously reach the least squares value once a circular self-closure constraint is imposed. As this adjustment is based on limited corresponding points, introducing a circular self-closure constraint instead of a single one is not likely to considerably increase computation time. Moreover, multi-view registration holding true to this constraint will safely converge to a global minimum.

## 4. Results and Discussion

### 4.1. Pair-Wise Registration Results

**Figure 5.**
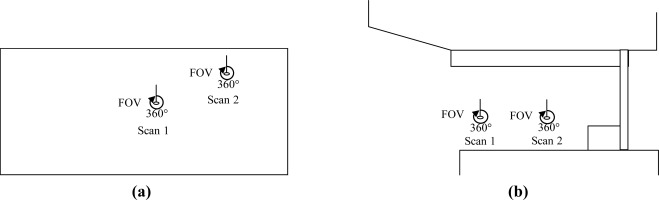

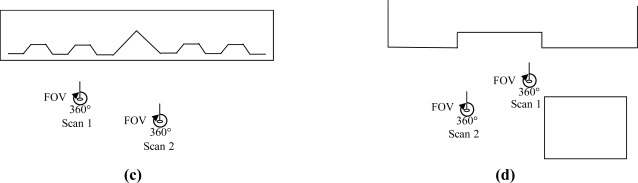
Floor plans of the test data sets. (a) Dataset 1; the scanned scene is the inside of an office. (b) Dataset 2; the scanned scene is the area between two buildings. (c) Dataset 3; the scanned scene is the area in front of the Aula Conference Centre of TU Delft. (d) Dataset 4; the scanned scene is an area surrounded by two buildings. The FOVs of the test datasets are 360° in all cases.

The proposed approach was tested with datasets acquired by a FARO LS 880 laser scanner, which can produce panoramic reflectance images. Dataset 1 contains 24 million TLS points for an office environment. Datasets 2 and 3, respectively, contain 33 million and 20 million TLS points for the Aula Conference Centre of TU Delft (Delft University of Technology), The Netherlands. Dataset 4 contains 26 million TLS points for a building on the TU Delft campus. Table 1 describes the test point clouds, and Figure 5 illustrates the relative locations of the scanner within the scenes. The scene ranges of the test datasets are 20, 77, 50 and 20 m, respectively. The same datasets were processed using the Leica HDS Cyclone 5.5 commercial software (www.leica-geosystems.com/hds/en/lgs_6515.htm) because it was available for our experiments. Cyclone 5.5 has a registration function to register point clouds using a point-to-surface based ICP in a semi-automatic way.

The angular resolution selected for Datasets 1 and 2 in both the horizontal and the vertical directions is a quarter of the full resolution given by the manufacturer, while the angular resolution of Datasets 3 and 4 is one-fifth of the full resolution.

Our experiments were conducted to evaluate the proposed approach using panoramic reflectance images from both the indoor and the outdoor scene. Our algorithm was implemented in C++. All of the tests were performed on a PC with an Intel Pentium IV CPU running at 3 GHz with 1 GB RAM.

Various error metrics have been defined to measure the registration accuracies of point clouds (e.g., [39]). In this paper, for the purpose of accuracy comparison, the distances between the corresponding points were measured. The use of indoor panoramic reflectance images for registration is explained in detail below.

As mentioned in Section 2.2, the panoramic reflectance images are generated in terms of the angular coordinates and reflectance value of each 3D point of the FARO LS 880 point clouds. Usually, the panoramic reflectance images are used to generate a photorealistic texture of the scanned object or surface. Similar to a black and white image, the user does not need much experience to interpret these data. Example applications of image matching and texture mapping using panoramic reflectance images can be found in the literature, as in construction analysis [40], tree species recognition [41], and TLS calibration [42].

**Figure 6.**
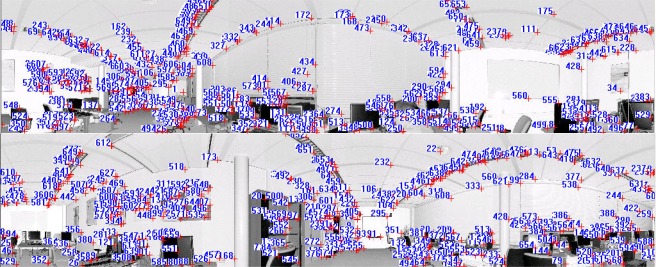
Corresponding points on two 360° reflectance images, which are identified by nearest neighbour search in terms of the Euclidean distance from the descriptor vector of the key point in the fixed image to that in the moving image. The numbers in the image are the indices of corresponding point pairs.

Figure 6 shows the panoramic reflectance images acquired by the FARO LS 880, in which pixel-to-point correspondence is inherent and there is no resolution difference between the reflectance image and the point cloud, as every pixel in the image has a unique corresponding 3D point in the point cloud. The corresponding 3D points are readily available in the data file.

We use the SIFT method to extract distinctive invariant features from the panoramic reflectance images and then identify the matches from the extracted features by looking for the descriptor vector with the closest Euclidean distance. The probability that a match is correct can be determined by taking the ratio of the distance from the closest neighbour to the distance of the second closest. This ratio was selected and used as a threshold for correspondence identification. The predefined threshold can be increased to select more matches or reduced to select only the most reliable match. We chose 0.8 as the threshold because experiments show that this can normally eliminate 90% of false matches [33]. As a result, a total of 655 corresponding point pairs were identified (see Figure 6). However, many of these point pairs were incorrectly accepted, which proved that the threshold of 0.8 is loose in this case. This loose threshold accepted many incorrect matches from the two panoramic reflectance images due to the repetitive patterns and salient changes in 3D viewpoint. To tackle this problem, we used the rigid geometric invariance derived from the TLS point clouds to prune the incorrect correspondences based on the TIN model. The topological relation derived from the TIN model was used to reduce the complexity of the distance invariance verification process for all of the corresponding points.

**Figure 7.**
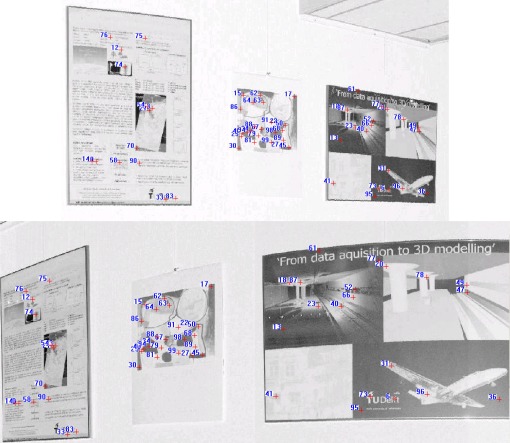
Corresponding points preserved after pruning from Dataset 1.

The threshold of distance invariance verification was defined before outlier detection. Equation 3 was used to compute σ_Δ*S*_ in terms of the angular resolution and angular and range accuracies listed in Table 1 for the distance invariance verification of each pair of correspondences. In order to provide good initial RTPs for the succeeding iterative process, we finally chose σ_Δ*S*_, rather than 3σ_Δ*S*_, as the threshold to remove outliers. As a result, only 110 correct correspondences were preserved, instead of the 655 correspondences shown in Figure 6. Figure 7 partly illustrates the correct correspondences. We used an iterative corresponding process to ensure the matching of a larger number of points and a reasonable distribution of the corresponding points to thereby include as many new matches as possible. During this process, the threshold became looser as 3σ_Δ*S*_ to accept more correct correspondences because most of the matching for point pairs became more accurate with increasing numbers of iterations.

**Figure 8.**
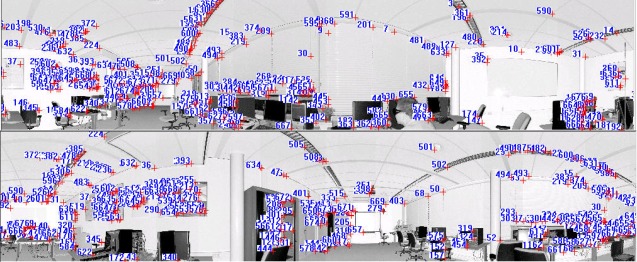
Corresponding points on the two reflectance images acquired after the iterative corresponding process. The numbers in the image are indices of corresponding point pairs.

As shown in Figure 8, 680 corresponding point pairs were generated after two iterations, and 99% of them were correct. The number of correct correspondences was almost 7 times that the number tracked based only on image information. Moreover, the distribution of 3D corresponding points was more reasonable.

Note: *n*_i_ is the total number of TLS points in scan *i* of Dataset 1, *iter* is the number of total iterations, rms is the root-mean-square error of the registration computed by least-squares adjustment, max, min, and avg are respectively the maximum, minimum and average distance between 3D corresponding point pairs that are selected as check point pairs after registration in a common coordinate frame.

**Figure 9.**
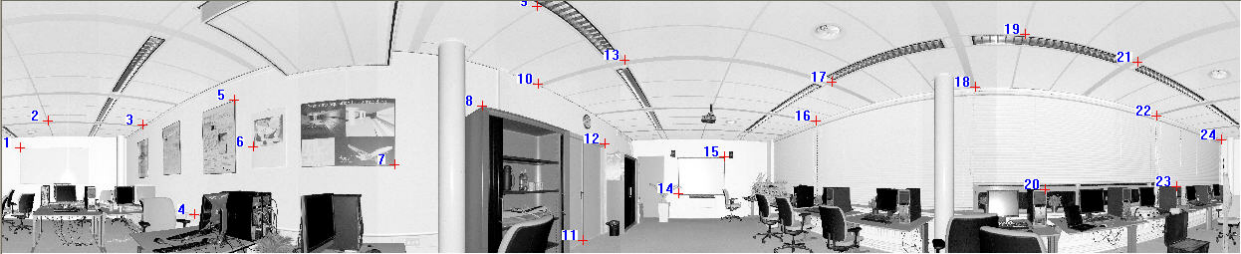
Check points used to measure registration accuracy. To ensure the proper distribution of check points, in each 60° segment of the 360° FOV four pairs of corresponding points were manually selected as check points in order to compare accuracies.

**Figure 10.**
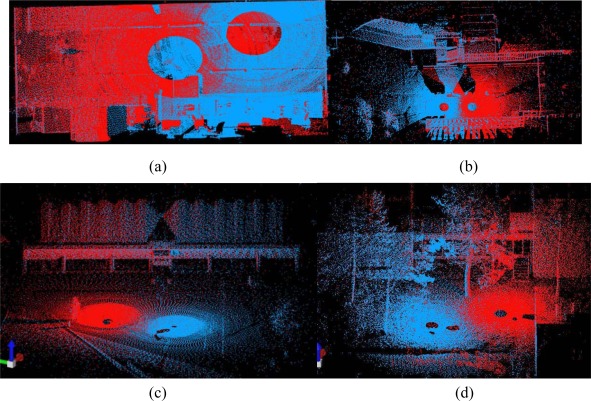
Pair-wise registered point clouds. (a) Dataset 1. (b) Dataset 2. (c) Dataset 3. (d) Dataset 4.

**Figure 11.**
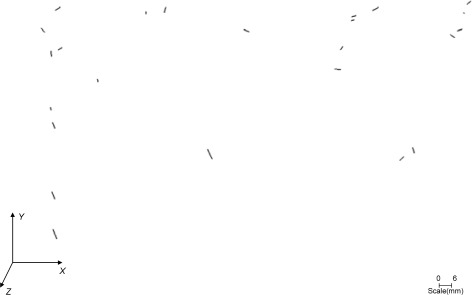
Scale image of the distances between 24 corresponding check points in the registered point cloud.

The registration of Dataset 1 was implemented with these correct corresponding points. In addition, 24 pairs of corresponding points were selected as check points for the purpose of accuracy comparison (see Figure 9). As a result, the registration accuracy and average distance between corresponding check points are in the order of millimetres, as shown in Table 2. Throughout the process, one iteration includes all of steps (2)–(5), as shown in Figure 1. The number of iterations may vary from dataset to dataset since the iterative process continues until no further accuracy improvement can be achieved. The computation time includes the entire iterative process illustrated in Figure 1. Processing of our method took 5 minutes, since the key point extraction and correspondence tracking on the panoramic reflectance image (image sizes of up to 12 million pixels) consume much of the computation time. Two scans of point clouds registered into a uniform coordinate frame are illustrated in Figure 10 (a). Figure 11 shows a scale image of the distances between 24 corresponding check points in the registered point cloud.

The registration function of Cyclone 5.5 using point-to-surface-based ICP was employed to implement the Dataset 1 registration. Compared to the results from our method, the accuracy is 2.4 mm; i.e., both of the accuracies are on the order of millimetres. The major difference is in the automation level and computational requirements. While the presented approach is completely automatic, the ICP requires a manual determination of initial corresponding point pairs. Moreover, the time cost of the whole process of ICP is 21 minutes, which is three times greater than that of our proposed method.

**Figure 12.**
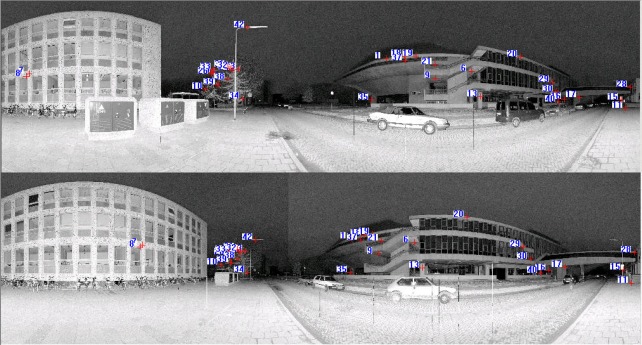
Corresponding points kept after pruning from Dataset 2.

**Figure 13.**
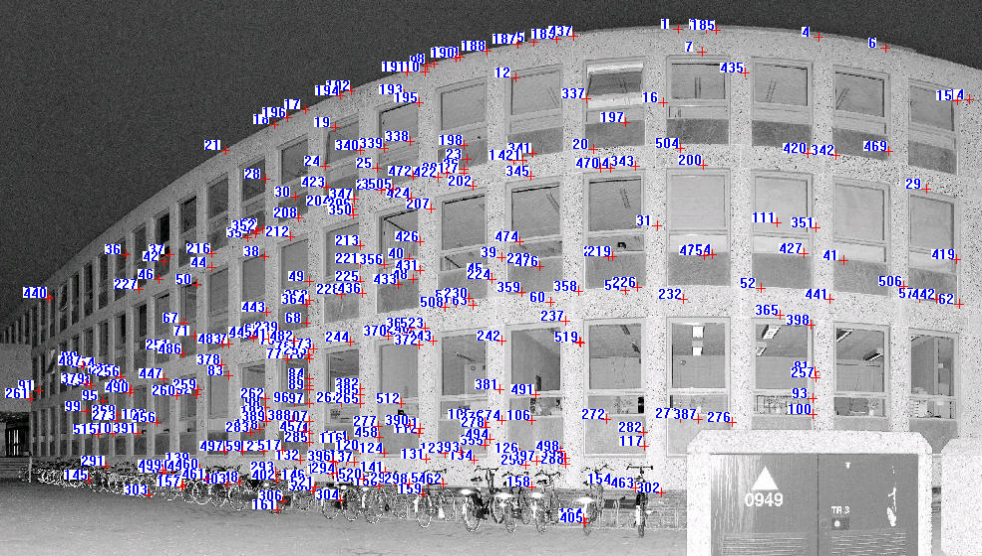

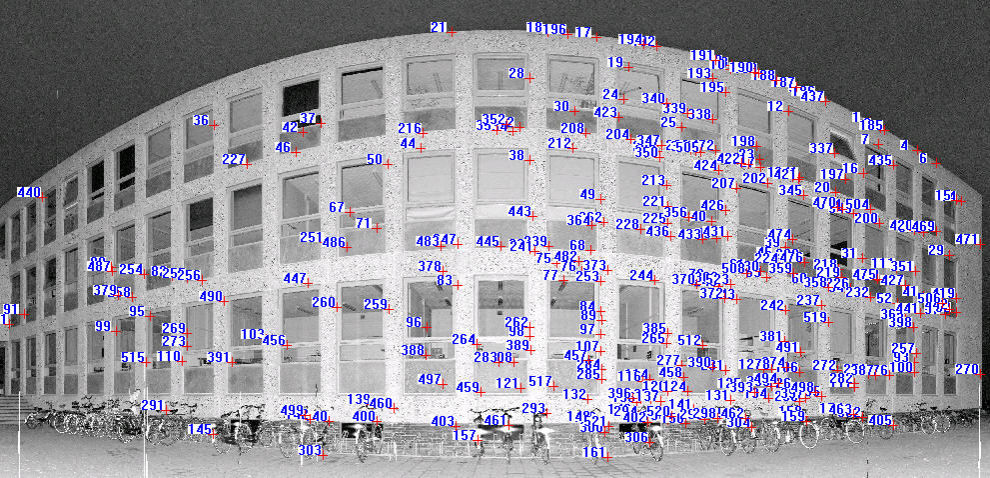
Evenly distributed corresponding points on the building facade after the iterative corresponding process.

Figure 5 (b) shows the floor plan of Dataset 2. The building facade exhibits repetitive patterns, with the result that few corresponding points on the facade persisted after pruning false matches (see Figure 12). Under the iterative matching process, plenty of correct corresponding point pairs on the facade were identified and the distribution of the matches becomes homogeneous in the panoramic reflectance images (see Figure 13). Twenty-four pairs of corresponding points were selected as check points for the purpose of accuracy comparisons. The registration result is also listed in Table 2. The registration accuracy is also on the order of millimetres, but is less than that of Dataset 1. We would expect this difference to contribute to the resolution difference between indoor and outdoor scenes. The whole process was completed in 6 minutes after only 2 iterations. As shown in Figure 10 (b), the registered point clouds are also well matched.

For comparison purposes, Datasets 3 and 4, which have lower angular resolutions, were also tested. The angular resolution selected for Datasets 3 and 4 is four-fifths of that of Datasets 1 and 2. Twenty-four pairs of corresponding points were selected as check points to measure the registration accuracy. The registration result listed in Table 2 shows that the registration accuracies of Datasets 3 and 4 are still on the order of millimetres, even though the angular resolution is reduced by one fifth. As a result, the registered point clouds are matched perfectly (see Figure 10 (c) and (d)).

ICP was also used to implement the outdoor dataset registration (i.e., Datasets 2–4). The registration accuracies are on the order of millimetres as well. However, the results of Datasets 2 and 4 were output after a few failures, as the selected corresponding point pairs failed to provide good initial parameters for registration. This result clearly shows that the implemented ICP approach is sensitive to the initial values.

### 4.2. Global Registration Results

Three bundle adjustment models were used in our study, named Model A (imposed by the circular self-closure constraint), Model B (imposed by the self-closure constraint of a single scan), and Model C (imposed by the last-to-first closure constraint of a single scan). All of the bundle adjustment models were implemented in C++ by the authors.

A total of 20 scans acquired by a FARO LS 880 laser scanner for the Aula Conference Centre of TU Delft were used to verify the three models. The method presented in Section 2 was used to implement pair-wise registration on these scans. Before the pair-wise registration, panoramic reflectance images were generated from the scans, and from each of them a key point database was extracted using SIFT. The corresponding scans for the pair-wise registration were automatically chosen by correspondence tracking among the key point databases. Table 3 lists the registration results.

Before global registration, the closure error from the last to the first scan was 5.1621 m, and the self-closure errors of the 20 scans ranged from 1.5460 to 6.0452 m, which suggests that although the residual error of each individual pair-wise registration was low, a propagation and an accumulation of the registration error are present such that large discrepancies exist between the last and first scans. The global registration was implemented based on the corresponding points and 20 sets of RTPs acquired from previous pair-wise registrations. The results of global registration are listed in Table 4. The highest accuracy was achieved by Model A. However, Model A is three times slower than Models B and C. This is because Model A allows for the inclusion of correspondences from all scans instead of a single one. Still, the computation cost is acceptable. Model C reduced the closure error from 5.1621 m to 0.0864 m after global registration, but it was still four times larger than that of Model A.

**Figure 14.**
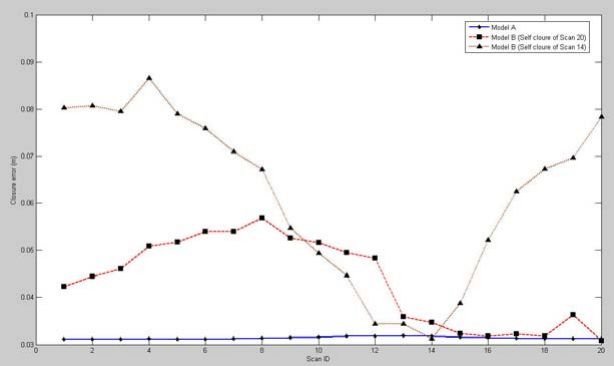
Closure errors of real scans after global registration.

As shown in Figure 14, Model B was tested by imposing the self-closure constraints derived from a single scan (e.g., Scans 14 and 20). With the determined transformation parameters, the closure error of each of the 20 scans was calculated. The closure errors of the same scan that result from imposing the self-closure constraints derived from the different scans. Moreover, the minimum of the closure errors in Model B was always the one from which the self-closure constraint was derived. By contrast, Model A enabled the closure error of all scans to simultaneously reach the minimum, which confirms that it may lead to the global optimisation result.

**Figure 15.**
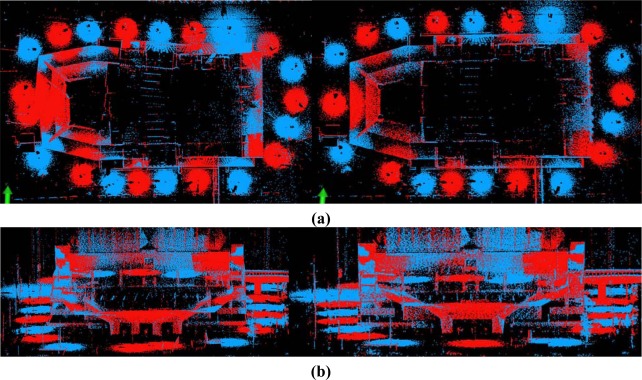
Registered real scans**. (a)** Top views: before (left) and after (right) global registration**. (b)** Front views: before (left) and after (right) global registration.

Figure 15 illustrates the registered point clouds before and after global registration. As with pair-wise registered point clouds, the adjacent scans are respectively coloured in red and blue to highlight the overlapping area.

The error accumulation before global registration is visualised by the twisting building shape (the left part of Figure 15(a)) and the four pillars (the left part of Figure 15 (b)) which are actually two. After the global registration using Model A, the 20 scans were registered with centimetre-scale accuracy by successfully eliminating the accumulated errors, as in the right part of Figure 15.

## 5. Summary and Outlook

In this paper, we have presented a fully automatic, iterative point cloud registration method based on panoramic reflectance imagery. This two-stage method consists of pair-wise and global registration. It makes use of the images to automatically find 2D correspondences and then generate 3D corresponding points from those correspondences. The 3D corresponding points can be used to compute RTPs, which are required for registering, using known pixel-to-point correspondences. The use of panoramic reflectance imagery, to which the pixel-to-point correspondence is inherent, realises the registration free of camera and camera-to-instrument calibrations and resolution differences between the images and the scans. Moreover, the panoramic reflectance imagery can be theoretically generated, in terms of the angular coordinate and reflectance value of each 3D point, directly from TLS scans. Rather than viewing correspondence tracking and the computation of the RTPs as a unilateral and ordinal procedure, we have integrated these two steps into an iterative process that allows for the optimisation of pair-wise registration, as it significantly improves the initial matches in three ways: (1) it removes outliers, (2) corrects false established correspondences, and (3) ensures a good distribution of 3D corresponding points in the overlapping regions. As for global registration, the self-closure constraint that forms is free of correspondence error. It can be extended to a circular model in order to minimise the self-closure errors across all scans through simultaneous least-squares adjustment.

Our tests with panoramic reflectance images show that the registration accuracy is of millimetre order and that the process is fully automatic and converges quickly. Since the registration uses only the corresponding points obtained by image matching, processing of the entire point clouds is avoided, which in turn significantly shortens the processing time. Furthermore, our method can compute RTPs in difficult cases, as for example when the normal vectors of the planes are parallel, which in turn may be problematic for primitive-based approaches.

The experiments with global registration with real TLS scan data prove that the closure error with respect to the adjustment model based on the last-to-first closure constraint consists of not only transformation error but also correspondence error. The results highlight that the bundle adjustment model imposed by the circular self-closure constraint may undoubtedly lead to the global optimised result.

Our future investigations will include efforts to improve the registration accuracy and the distance invariance error. Laser scanner accuracy (e.g., incident angle, edge effects) will be investigated to estimate the distance invariance error. In addition, the proposed method will be extended to an integrated change detection/registration approach for TLS applications in emergency response.

## Figures and Tables

**Table 1.** Description of the test point clouds.

**Point cloud**	**Scan/Image Angular resolution**	**Angular accuracy**	**Range accuracy**
Datasets 1 and 2	0.036°	±0.009°	±3 mm
Datasets 3 and 4	0.045°	±0.009°	±3 mm

**Table 2.** Results of the pair-wise registration.

**Proposed method**	***n*_1_** ***n*_2_**	***iter***	**rms (m)**	**max (m)**	**min (m)**	**avg (m)**	**Time (min)**
Dataset 1	11987424	2	0.0037	0.0053	0.0009	0.0035	5.0
	11974976						
Dataset 2	16726500	2	0.0044	0.0145	0.0018	0.0066	6.0
	16713375						
Dataset 3	10006564	3	0.0038	0.0185	0.0025	0.0078	5.5
	10046768						
Dataset 4	13266288	4	0.0039	0.0083	0.0010	0.0041	6.3
	13259400						

**Table 3.** Results of the pair-wise registration of 20 scans.

**Proposed method**	***iter***	**rms (m)**	**time (min)**	**Proposed method**	***iter***	**Rms (m)**	**time (min)**

Scan 2-1	3	0.0058	5.4	Scan 12-11	3	0.0073	5.3
Scan 3-2	3	0.0067	5.5	Scan 13-12	5	0.0098	6.6
Scan 4-3	4	0.0079	5.9	Scan 14-13	5	0.0085	6.7
Scan 5-4	5	0.0074	6.7	Scan 15-14	3	0.0023	5.3
Scan 6-5	4	0.0036	6.1	Scan 16-15	4	0.0075	6.1
Scan 7-6	4	0.0082	6.0	Scan 17-16	4	0.0056	6.1
Scan 8-7	5	0.0098	6.6	Scan 18-17	5	0.0069	6.8
Scan 9-8	5	0.0095	6.7	Scan 19-18	4	0.0039	6.0
Scan 10-9	4	0.0037	6.2	Scan 20-19	3	0.0015	5.4
Scan 11-10	4	0.0051	6.0	Scan 1–20	5	0.0079	6.7

**Table 4.** Results of global registration.

**Model**	***iter***	**RMS (m)**	**Time (min)**

A	6	0.0340	1.98
B (Scan 20)	4	0.0351	0.62
B (Scan 14)	4	0.0352	0.62
C	4	0.0381	0.63
